# Supplementary material for: An IMiD-induced SALL4 degron system for selective degradation of target proteins
Source: Commun Biol. 2020 Sep 18;3:515. doi: 10.1038/s42003-020-01240-5 (PMC7501283; doi:10.1038/s42003-020-01240-5)

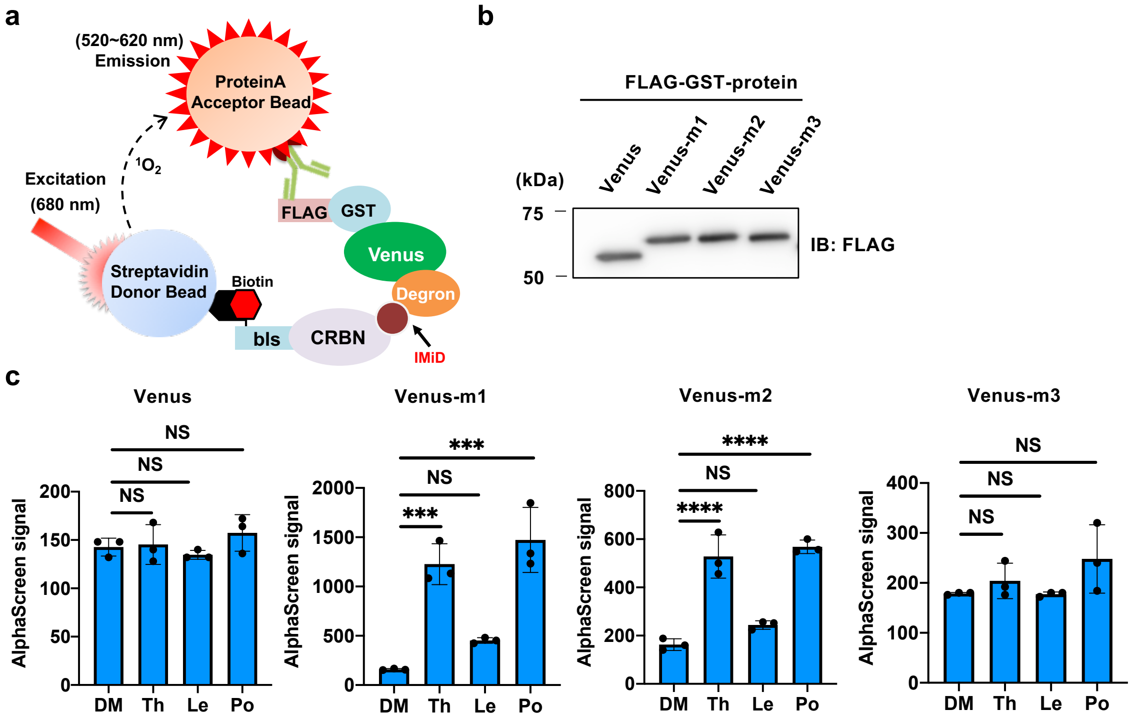

Supplementary Fig. 1 *In vitro* binding assay using AlphaScreen technology.

a, Schematic diagram of the IMiD-dependent *in vitro* binding assay between CRBN and Venus-SALL4 degron using AlphaScreen technology. **b,** Immunoblot analysis of protein synthesis of Venus-SALL4 degron using the wheat cell-free system. **c,** *In vitro* binding assay for thalidomide, lenalidomide, and pomalidomide. Interactions between bls-CRBN and FLAG-GST-Venus, FLAG-GST-Venus-m1, -m2, or -m3 in the presence of DMSO (DM), 50 µM thalidomide (Th), lenalidomide (Le), or pomalidomide (Po) were analyzed using AlphaScreen technology. Error bars indicate the mean ± SD (n = 3), and *P*-values were calculated by one-way ANOVA with Tukey's post-hoc tests (NS = not significant; $^{***}P < 0.001$ and $^{****}P < 0.0001$.

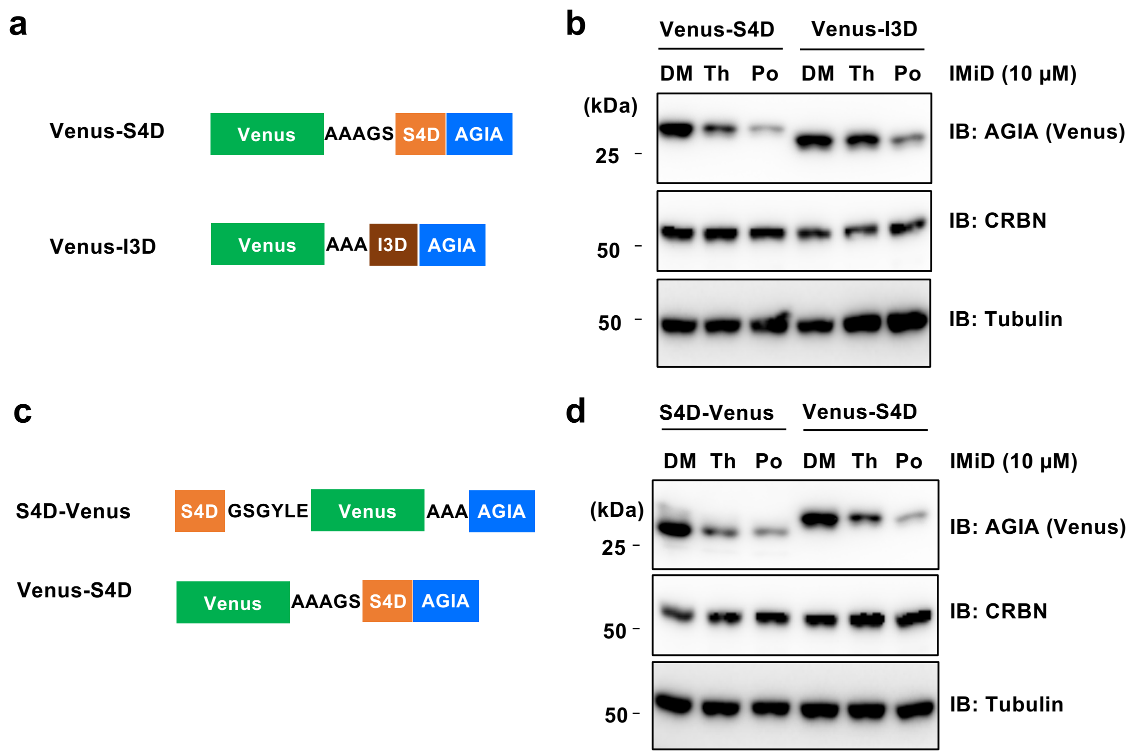

Supplementary Fig. 2 Comparison of the tag position in IKZF3 degron (I3D) and S4D.

a, Schematic diagram of the ORFs of the expression vectors encoding Venus-S4D and Venus-I3D. **b,** Immunoblot analysis of Venus-S4D and Venus-I3D. HEK293T cells expressing Venus-S4D-AGIA or Venus-I3D-AGIA and FLAG-CRBN were treated with DMSO (DM), thalidomide (Th), or pomalidomide (Po) for 16 h. **c,** Schematic diagram of the ORFs of the expression vectors encoding S4D-Venus and Venus-S4D. **d,** Immunoblot analysis of the protein level of S4D-Venus and Venus-S4D. HEK293T cells expressing S4D-Venus-AGIA or Venus-S4D-AGIA were treated with DM, Th, or Po for 16 h.

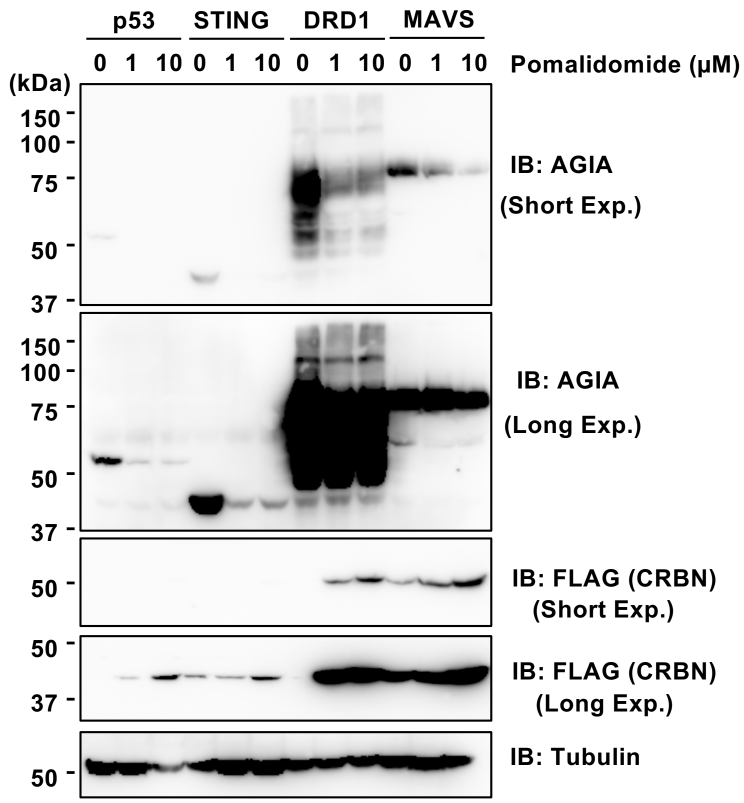

Supplementary Fig. 3 IMiD-induced degradation of S4D-tagged proteins in CRBN$^{-/-}$ HEK293T.

a, Immunoblot analysis of the dose-dependent degradation of proteins with various subcellular localizations. CRBN$^{-/-}$ HEK293T cells expressing p53-S4D-AGIA, STING-S4D-AGIA, DRD1-S4D-AGIA, or MAVS-S4D-AGIA and FLAG-CRBN were treated with DMSO or pomalidomide for 16 h.

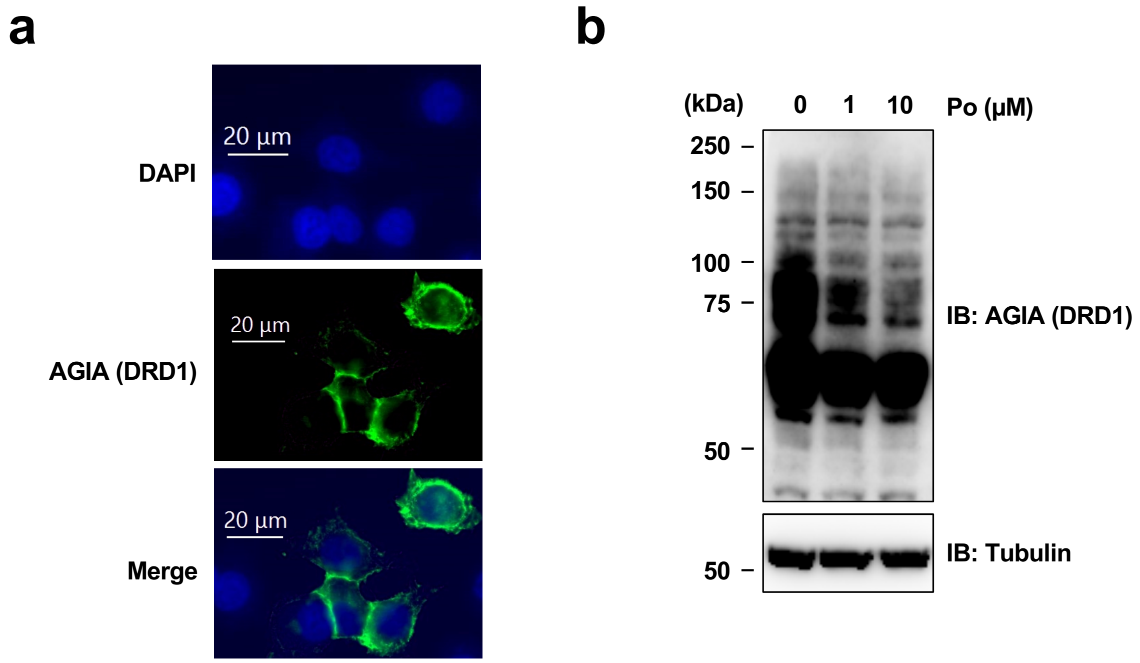

Supplementary Fig. 4 Localization and degradation of stably expressed DRD1-S4D-AGIA in HeLa cells.

a, Localization of stably expressed DRD1-S4D-AGIA by immunofluorescent staining in HeLa cells. Scale bars, 20 µm. **b,** Immunoblot analysis of HeLa cells expressing DRD1-S4D-AGIA stably were treated with DMSO or pomalidomide (Po) in the presence of 24 h.

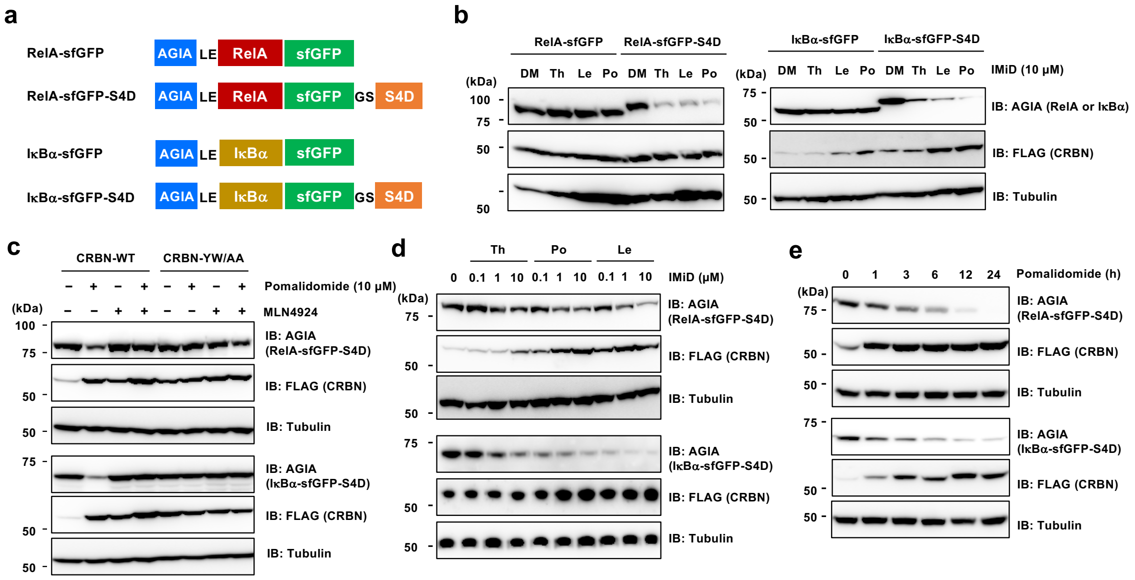

Supplementary Fig. 5 Degradation of S4D-tagged RelA or IκBα transiently expressed in CRBN$^{-/-}$ HEK293T cells.

a, Schematic diagram of the ORFs of the expression vectors encoding RelA-sfGFP, RelA-sfGFP-S4D, IκBα-sfGFP, and IκBα-sfGFP-S4D. **b,** Immunoblot analysis of S4D-tagged RelA and IκBα. CRBN$^{-/-}$ HEK293T cells expressing AGIA-tagged RelA-sfGFP, RelA-sfGFP-S4D, IκBα-sfGFP, or IκBα-sfGFP-S4D and FLAG-CRBN were treated with DMSO (DM), thalidomide (Th), lenalidomide (Le), or pomalidomide (Po) for 16 h. **c,** Immunoblot analysis of CRBN$^{-/-}$ HEK293T cells expressing AGIA-RelA-sfGFP-S4D or -IκBα-sfGFP-S4D and FLAG-CRBN-WT or FLAG-CRBN-YW/AA treated with DMSO or 10 μM pomalidomide in the presence of DMSO or 2 μM MLN4924 for 9 h. **d,** Dose-dependent degradation of S4D-tagged RelA and IκBα. CRBN$^{-/-}$ HEK293T cells expressing AGIA-RelA-sfGFP-S4D or -IκBα-sfGFP-S4D and FLAG-CRBN were treated with DMSO, thalidomide, lenalidomide, or pomalidomide at the indicated concentrations and analyzed by immunoblot. **e,** Time-dependent degradation of S4D-tagged RelA and IκBα. CRBN$^{-/-}$ HEK293T cells expressing AGIA-RelA-sfGFP-S4D or -IκBα-sfGFP-S4D and FLAG-CRBN were treated with DMSO or 10 μM pomalidomide for the indicated times and were analyzed by immunoblot.

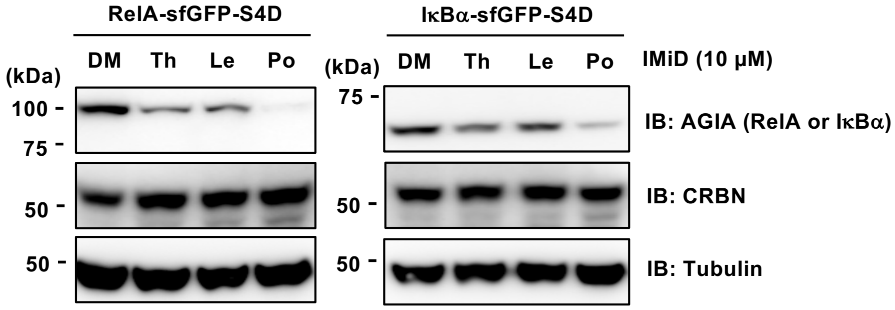

Supplementary Fig. 6 Protein degradation of S4D-tagged RelA and IκBα by endogenous CRBN in HeLa cells.

a, Immunoblot analysis of S4D-tagged RelA and IκBα in HeLa cells. HeLa cells expressing AGIA-RelA-sfGFP-S4D or -IκBα-sfGFP-S4D were treated with DMSO (DM), thalidomide (Th), lenalidomide (Le), or pomalidomide (Po) for 16 h.

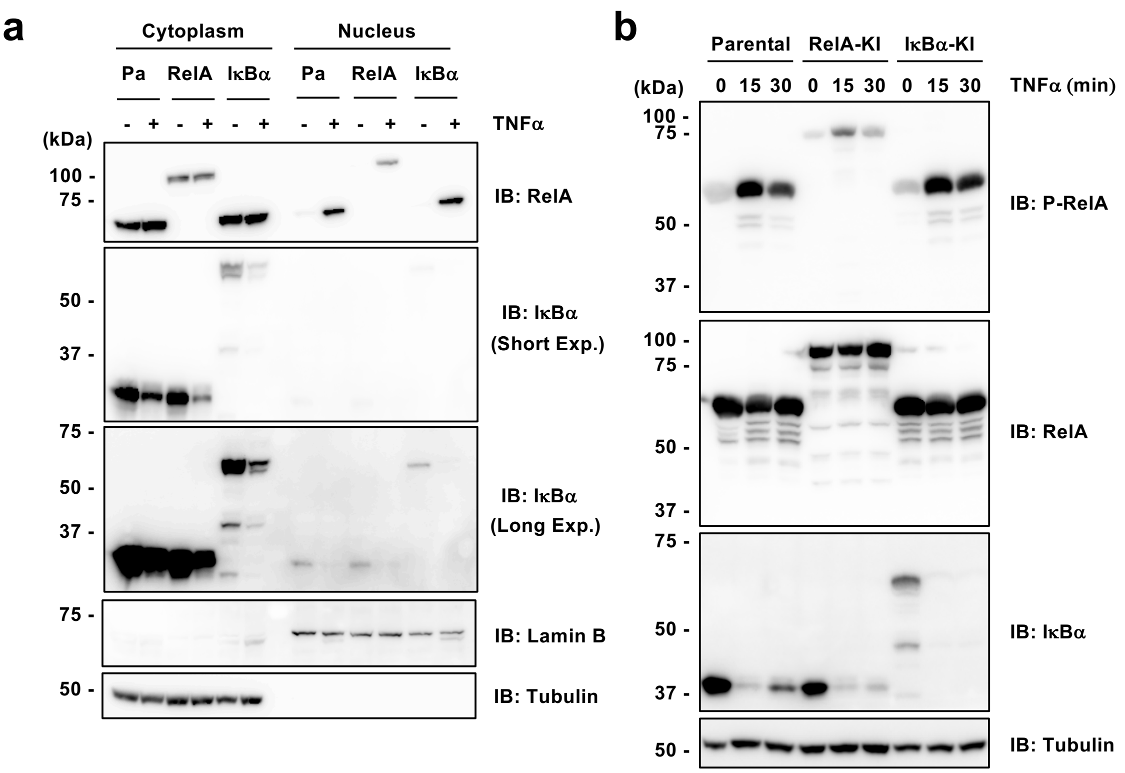

Supplementary Fig. 7 Responses to TNF-α stimulation in S4D-tagged RelA and IκBα knock-in cells.

a, Nuclear translocation of RelA in TNF-α-stimulated parental (Pa) and KI HeLa cells. Pa, IκBα-KI (IκBα), or RelA-KI (RelA) cells were stimulated with 20 ng/ml TNF-α for 15 min, and the nuclear and cytoplasmic extracts were analyzed by immunoblot. **b,** Immunoblot analysis of the phosphorylation and degradation of components of NF-κB signal transduction. Parental or KI cells were treated with 20 ng/ml TNF-α for the indicated times, and the lysates were analyzed by immunoblot.

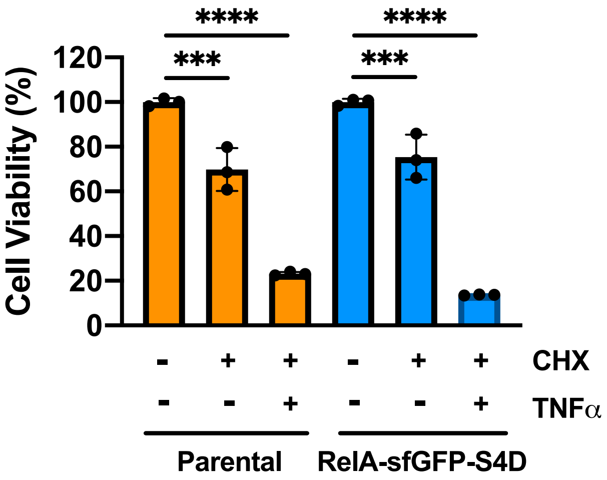

Supplementary Fig. 8 TNF-α and CHX treatment reduced cell viability both in the parental and RelA-sfGFP-S4D KI cells.

Accelerated cell death by TNF-α and CHX treatment. Parental and RelA-sfGFP-S4D-KI cells were treated with CHX or CHX plus TNF-α for 24 h, and the cell viabilities were measured by MTS assay. Error bars represent the mean ± SD (n = 3), and *P*-values were calculated by one-way ANOVA with Tukey's post-hoc test ($^{***}P < 0.001$ and $^{****}P < 0.0001$).

Uncropped blot data

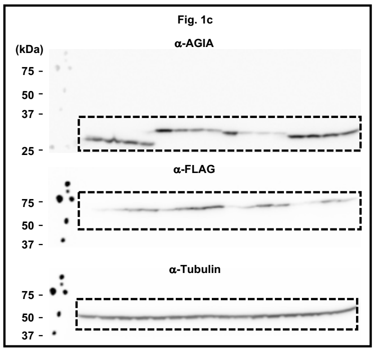
Fig. 1c
(kDa)
α-AGIA
75
50
37
25
α-FLAG
75
50
37
α-Tubulin
75
50
37

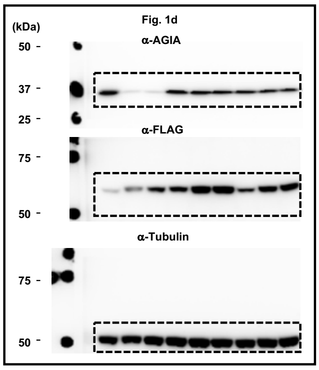
Fig. 1d
(kDa)
α-AGIA
50
37
25
α-FLAG
75
50
α-Tubulin
75
50

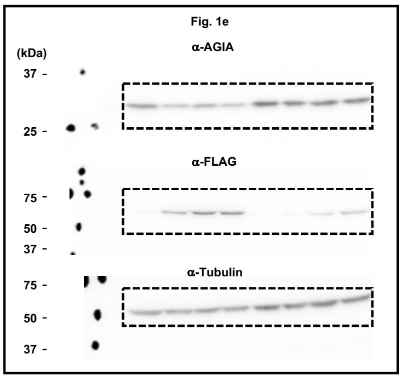
Fig. 1e
(kDa)
α-AGIA
37
25
α-FLAG
75
50
37
α-Tubulin
75
50
37

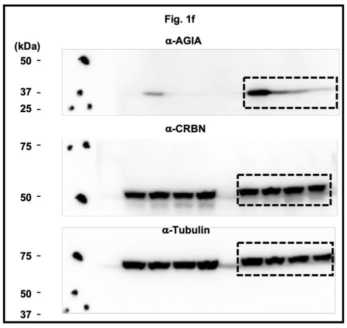
Fig. 1f
(kDa)
α-AGIA
50
37
25
α-CRBN
75
50
α-Tubulin
75
50
37

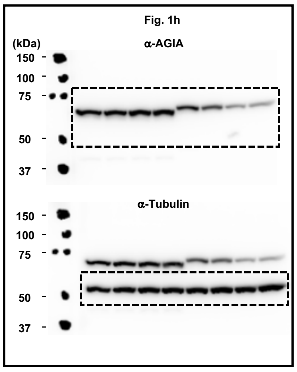
Fig. 1h
(kDa)
α-AGIA
150
100
75
50
37
α-Tubulin
150
100
75
50
37

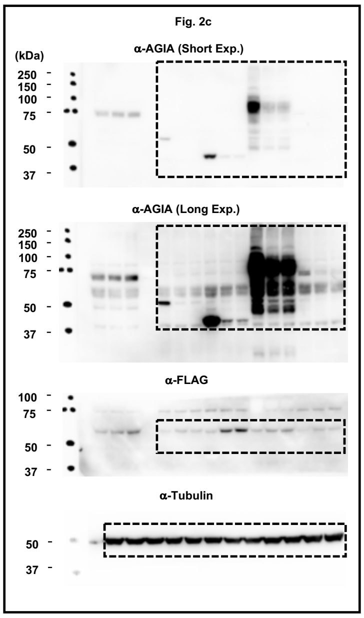
Fig. 2c
(kDa)
α-AGIA (Short Exp.)
250
150
100
75
50
37
α-AGIA (Long Exp.)
250
150
100
75
50
37
α-FLAG
100
75
50
37
α-Tubulin
50
37

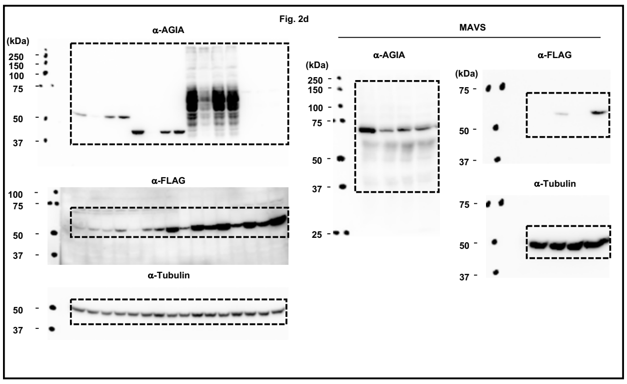
Fig. 2d
(kDa)
α-AGIA
250
150
100
75
50
37
α-FLAG
100
75
50
37
α-Tubulin
50
37
MAVS
(kDa)
α-AGIA
250
150
100
75
50
37
25
(kDa)
α-FLAG
75
50
37
α-Tubulin
75
50
37

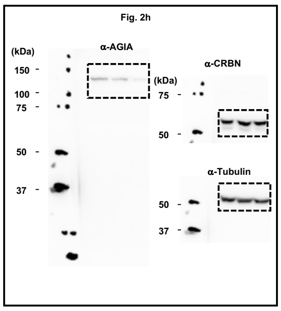
Fig. 2h
(kDa)
α-AGIA
150
100
75
50
37
(kDa)
α-CRBN
75
50
α-Tubulin
50
37

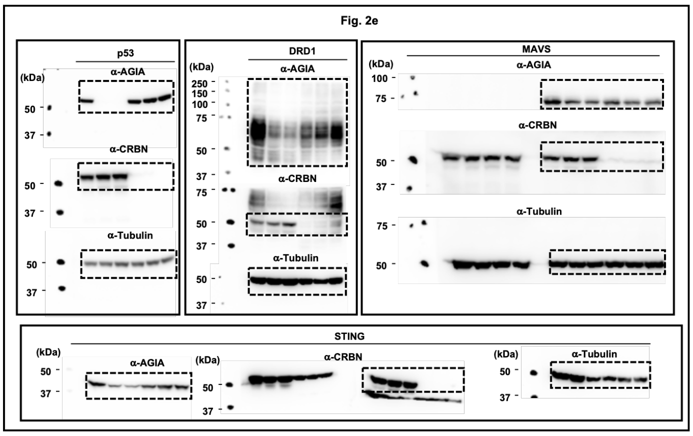
Fig. 2e
p53
(kDa)
α-AGIA
50
37
α-CRBN
50
37
α-Tubulin
50
37
DRD1
(kDa)
α-AGIA
250
150
100
75
50
37
α-CRBN
75
50
37
α-Tubulin
50
37
MAVS
(kDa)
α-AGIA
100
75
α-CRBN
50
37
α-Tubulin
75
50
STING
(kDa)
α-AGIA
50
37
(kDa)
α-CRBN
50
37
(kDa)
α-Tubulin
50
37

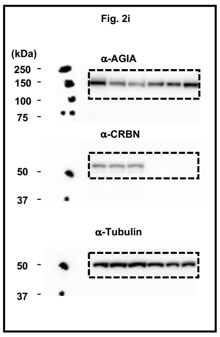
Fig. 2i
(kDa)
α-AGIA
250
150
100
75
α-CRBN
50
37
α-Tubulin
50
37

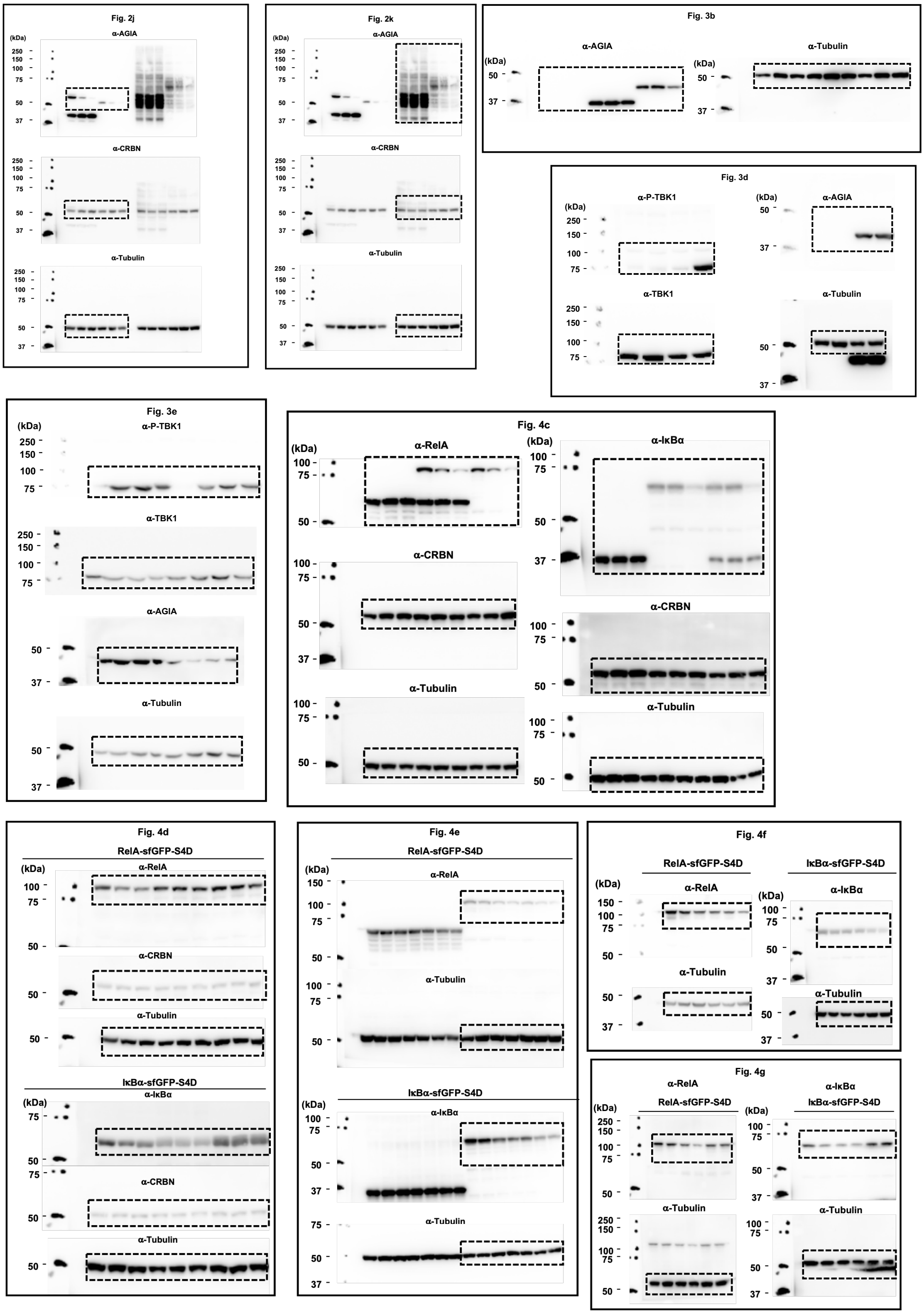
Fig. 2j
α-AGIA
α-CRBN
α-Tubulin
Fig. 2k
α-AGIA
α-CRBN
α-Tubulin
Fig. 3b
α-AGIA
α-Tubulin
Fig. 3d
α-P-TBK1
α-AGIA
α-TBK1
α-Tubulin
Fig. 3e
α-P-TBK1
α-TBK1
α-AGIA
α-Tubulin
Fig. 4c
α-RelA
α-IκBα
α-CRBN
α-CRBN
α-Tubulin
α-Tubulin
Fig. 4d
RelA-sfGFP-S4D
α-RelA
α-CRBN
α-Tubulin
IκBα-sfGFP-S4D
α-IκBα
α-CRBN
α-Tubulin
Fig. 4e
RelA-sfGFP-S4D
α-RelA
α-Tubulin
IκBα-sfGFP-S4D
α-IκBα
α-Tubulin
Fig. 4f
RelA-sfGFP-S4D
IκBα-sfGFP-S4D
α-RelA
α-IκBα
α-Tubulin
α-Tubulin
Fig. 4g
α-RelA
RelA-sfGFP-S4D
α-IκBα
IκBα-sfGFP-S4D
α-Tubulin
α-Tubulin
(kDa)

Fig. 5d

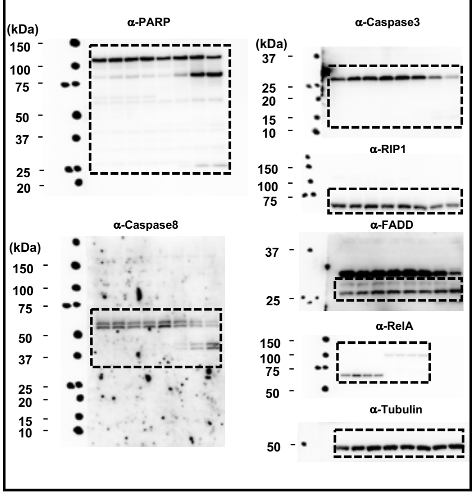

Fig. 5f

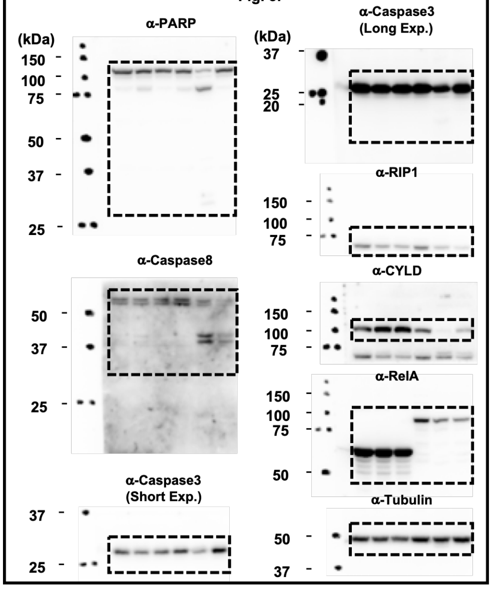

Fig. 6a

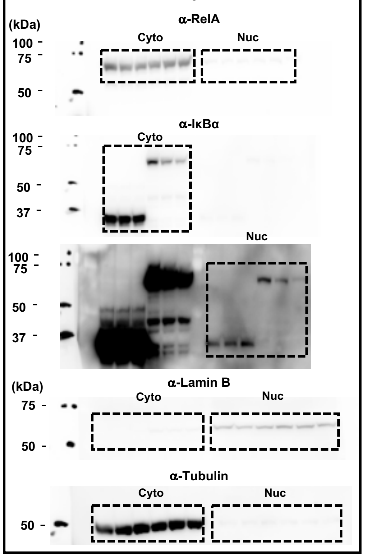

Fig. 6b

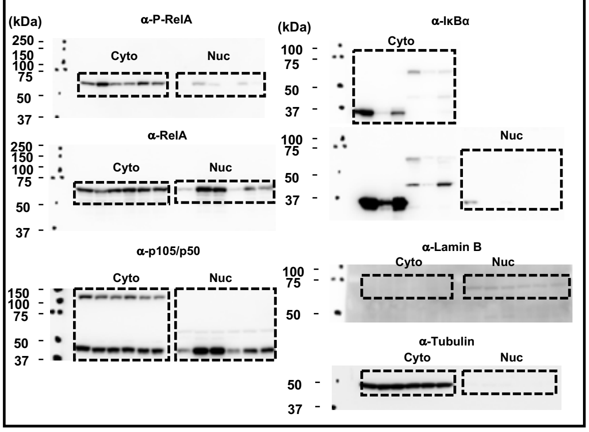

Fig. 7e

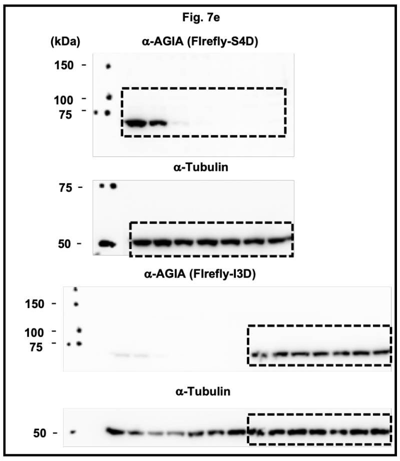

Fig. 7f

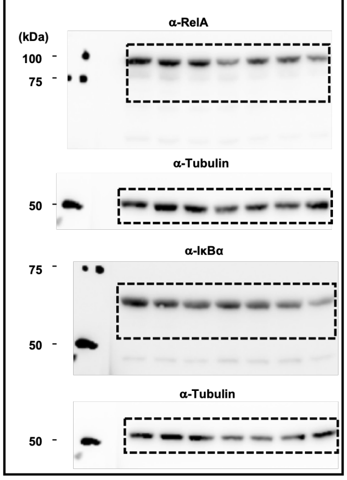

Supplement: Supplementary file 1 — Supplementary Information [file 42003_2020_1240_MOESM1_ESM.pdf]
