# Supplementary material for: An IMiD-induced SALL4 degron system for selective degradation of target proteins
Source: Commun Biol. 2020 Sep 18;3:515. doi: 10.1038/s42003-020-01240-5 (PMC7501283; doi:10.1038/s42003-020-01240-5)
Supplement: Supplementary file 2 — Description of Additional Supplementary Files [file 42003_2020_1240_MOESM2_ESM.pdf]

Descriptions of Additional Supplementary Files

Supplementary Data 1: source data file
