# Supplementary material for: An IMiD-induced SALL4 degron system for selective degradation of target proteins
Source: Commun Biol. 2020 Sep 18;3:515. doi: 10.1038/s42003-020-01240-5 (PMC7501283; doi:10.1038/s42003-020-01240-5)
Supplement: Supplementary file 5 — Reporting Summary [file 42003_2020_1240_MOESM5_ESM.pdf]

nature research

Corresponding author(s): Tatsuya Sawasaki

Last updated by author(s): Jul 13, 2020

Reporting Summary

Nature Research wishes to improve the reproducibility of the work that we publish. This form provides structure for consistency and transparency in reporting. For further information on Nature Research policies, see our Editorial Policies and the Editorial Policy Checklist.

Statistics

For all statistical analyses, confirm that the following items are present in the figure legend, table legend, main text, or Methods section.

n/a	Confirmed	
☐	☒	The exact sample size (n) for each experimental group/condition, given as a discrete number and unit of measurement
☐	☒	A statement on whether measurements were taken from distinct samples or whether the same sample was measured repeatedly
☐	☒	The statistical test(s) used AND whether they are one- or two-sided<br>*Only common tests should be described solely by name; describe more complex techniques in the Methods section.*
☒	☐	A description of all covariates tested
☒	☐	A description of any assumptions or corrections, such as tests of normality and adjustment for multiple comparisons
☐	☒	A full description of the statistical parameters including central tendency (e.g. means) or other basic estimates (e.g. regression coefficient) AND variation (e.g. standard deviation) or associated estimates of uncertainty (e.g. confidence intervals)
☐	☒	For null hypothesis testing, the test statistic (e.g. F, t, r) with confidence intervals, effect sizes, degrees of freedom and P value noted<br>*Give P values as exact values whenever suitable.*
☒	☐	For Bayesian analysis, information on the choice of priors and Markov chain Monte Carlo settings
☒	☐	For hierarchical and complex designs, identification of the appropriate level for tests and full reporting of outcomes
☒	☐	Estimates of effect sizes (e.g. Cohen's d, Pearson's r), indicating how they were calculated

Our web collection on statistics for biologists contains articles on many of the points above.

Software and code

Policy information about availability of computer code

Data collection	All data collection in this study were performed using softwares attached to each detector.<br>Image Quant LAS 4000 software (GE Healthcare) for immunoblot.<br>Wallac Envision Manager software (ParkinElmer) for AlphaScreen.<br>LightCycler 96 software (Roche) for quantitative-PCR.<br>SpectlraMax M3 microplate reader (Molecular Devises) for MTS assay.<br>BZ-X810 Microscope (Keyence) for immunofluorescence staining.
Data analysis	Image analysis was performed using ImageJ software.<br>Data analysis and significant changes were performed using Excel or GraphPad Prism 8.

For manuscripts utilizing custom algorithms or software that are central to the research but not yet described in published literature, software must be made available to editors and reviewers. We strongly encourage code deposition in a community repository (e.g. GitHub). See the Nature Research guidelines for submitting code & software for further information.

Data

Policy information about availability of data

All manuscripts must include a data availability statement. This statement should provide the following information, where applicable:

- Accession codes, unique identifiers, or web links for publicly available datasets
- A list of figures that have associated raw data
- A description of any restrictions on data availability

All data are included in Supplementary Data 1 and Supplementary information files.

Field-specific reporting

Please select the one below that is the best fit for your research. If you are not sure, read the appropriate sections before making your selection.

☒ Life sciences ☐ Behavioural & social sciences ☐ Ecological, evolutionary & environmental sciences

For a reference copy of the document with all sections, see nature.com/documents/nr-reporting-summary-flat.pdf

Life sciences study design

All studies must disclose on these points even when the disclosure is negative.

Sample size	No statistical analysis was performed to determine sample size. Sample sizes were determined based on previous studies in the field to enable statistical analyses and ensure reproducibility.
Data exclusions	No data were excluded from analysis.
Replication	All experiments were performed in two or more replicates.
Randomization	No randomization was used in this study as it is not necessary in this experiments.
Blinding	Blinding was not conducted in this study.

Reporting for specific materials, systems and methods

We require information from authors about some types of materials, experimental systems and methods used in many studies. Here, indicate whether each material, system or method listed is relevant to your study. If you are not sure if a list item applies to your research, read the appropriate section before selecting a response.

Materials & experimental systems

n/a	Involved in the study	
☐	☒	Antibodies
☐	☒	Eukaryotic cell lines
☒	☐	Palaeontology and archaeology
☒	☐	Animals and other organisms
☒	☐	Human research participants
☒	☐	Clinical data
☒	☐	Dual use research of concern

Methods

n/a	Involved in the study	
☒	☐	ChIP-seq
☒	☐	Flow cytometry
☒	☐	MRI-based neuroimaging

Antibodies

Antibodies used	Anti-FLAG mouse mAb (HRP-conjugated, Sigma-Aldrich, #A8592, 1:5000) and anti-AGIA rabbit mAb (HRP-conjugated, produced in our laboratory, 1:10000) were used to detect epitope-tagged proteins. Anti-α-tubulin rabbit pAb (HRP-conjugated, MBL, #PM054-7, 1:10000) was used to detect α-tubulin. Anti-CRBN rabbit mAb (Cell Signaling Technology, #71810, 1:1000), anti-IκBα mouse mAb (Cell Signaling Technology, #4814, 1:1000), anti-NF-κB1 p105/p50 rabbit mAb (Cell Signaling Technology, #12540, 1:1000), anti-p65/RelA rabbit mAb (Cell Signaling Technology, #8242, 1:1000), phospho-p65/RelA rabbit mAb (Cell Signaling Technology, #3033, 1:1000), anti-CYLD rabbit mAb (Cell Signaling Technology, #8462, 1:1000), anti-RIP1 rabbit mAb (Cell Signaling Technology, #3493, 1:1000), anti-Caspase-8 mouse mAb (Cell Signaling Technology, #9746, 1:1000), anti-Caspase-3 rabbit Ab (Cell Signaling Technology, #9662, 1:1000), anti-PARP rabbit Ab (Cell Signaling Technology, #9542, 1:1000), anti-phospho-TBK1 rabbit mAb (Cell Signaling Technology, #5483, 1:1000), anti-TBK1 rabbit mAb (Cell Signaling Technology, #3504, 1:1000), and anti-laminB goat pAb (Santa Cruz Biotechnology, #sc-6217, 1:500) were used as primary antibody. Anti-rabbit IgG (HRP-conjugated, Cell Signaling Technology, #7074, 1:10000), anti-mouse IgG (HRP-conjugated, Cell Signaling Technology, #7076, 1:10000), anti-goat IgG (HRP-conjugated, Invitrogen/ Thermo Fisher Scientific, #81-1620, 1:10000) for immunoblot. Anti-AGIA rabbit mAb (produced in our laboratory, 1:1000), anti-GM130 mouse mAb (MBL, #M179-3MS, 1:200), anti-rabbit IgG Alexa Fluor 488 (Invitrogen/Thermo Fisher Scientific, #A-11008, 1:1000), and Anti-mouse IgG Alexa Fluor 555 (Invitrogen/Thermo Fisher Scientific, #A-21422, 1:1000) were used for immunofluorescence staining.
Validation	All primary antibodies in this study were purchased from commercial companies. All of these antibodies were stated to be able to detect each endogenous protein in supplier's datasheets and these antibodies were used according to supplier's protocol. AGIA antibody has been validated in published paper (Yano, et al., PLoS ONE 11: e0156716). cited in Methods.

Eukaryotic cell lines

Policy information about cell lines

Cell line source(s)	HEK293T and HeLa cells were purchased from Riken BioResource Research Center (Riken BRC).
Authentication	All cell lines used in this study are cell lines authenticated by each manufacturer.
Mycoplasma contamination	All cell lines used in this study were tested negative for mycoplasma.
Commonly misidentified lines (See ICLAC register)	No commonly misidentified cell lines were used in this study.
